# A randomized, placebo-controlled study of the cardiovascular safety of the once-weekly DPP-4 inhibitor omarigliptin in patients with type 2 diabetes mellitus

**DOI:** 10.1186/s12933-017-0593-8

**Published:** 2017-09-11

**Authors:** Ira Gantz, Menghui Chen, Shailaja Suryawanshi, Catherine Ntabadde, Sukrut Shah, Edward A. O’Neill, Samuel S. Engel, Keith D. Kaufman, Eseng Lai

**Affiliations:** 1 Merck & Co., Inc., Kenilworth, NJ USA; 2 Merck & Co., Inc., RY34-A260, P.O. Box 2000, Rahway, NJ 07065 USA; 3 Merck & Co., Inc., RY34-A304, P.O. Box 2000, Rahway, NJ 07065 USA; 4 Merck & Co., Inc., RY34-A314, P.O. Box 2000, Rahway, NJ 07065 USA; 5 Merck & Co., Inc., RY34A-228, P.O. Box 2000, Rahway, NJ 07065 USA; 6 Merck & Co., Inc., RY34-A578, P.O. Box 2000, Rahway, NJ 07065 USA; 7 Merck & Co., Inc., RY34-A232, P.O. Box 2000, Rahway, NJ 07065 USA; 8 Merck & Co., Inc., RY34-A248, P.O. Box 2000, Rahway, NJ 07065 USA; 9 Merck & Co., Inc., RY34-A500, P.O. Box 2000, Rahway, NJ 07065 USA

**Keywords:** Antihyperglycemic agent, Dipeptidyl peptidase-4, Incretin, MK-3102

## Abstract

**Background:**

Omarigliptin is a once-weekly (q.w.) oral DPP-4 inhibitor that is approved for the treatment of patients with type 2 diabetes mellitus (T2DM) in Japan. To support approval of omarigliptin in the United States, the clinical development program included a cardiovascular (CV) safety study. Subsequently, a business decision was made not to submit a marketing application for omarigliptin in the United States, and the CV safety study was terminated. Herein we report an analysis of data from that early-terminated study.

**Methods:**

In this randomized, double-blind study, 4202 patients with T2DM and established CV disease were assigned to either omarigliptin 25 mg q.w. or matching placebo in addition to their existing diabetes therapy. A Cox proportional hazards model was used to summarize the primary endpoint of time to first major adverse CV event (MACE, the composite of CV death, nonfatal myocardial infarction, and nonfatal stroke) and the analysis of first event of hospitalization for heart failure (hHF).

**Results:**

The median follow-up was approximately 96 weeks (range 1.1–178.6 weeks). The primary MACE outcome occurred in 114/2092 patients in the omarigliptin group (5.45%; 2.96/100 patient-years) and 114/2100 patients in the placebo group (5.43%; 2.97/100 patient-years), with a hazard ratio (HR) of 1.00 (95% confidence interval [CI] 0.77, 1.29). The hHF outcome occurred in 20/2092 patients in the omarigliptin group (0.96%; 0.51/100 patient-years) and 33/2100 patients in the placebo group (1.57%; 0.85/100 patient-years), with an HR of 0.60 (95% CI 0.35, 1.05). After 142 weeks, the least-squares mean difference (omarigliptin vs. placebo) in glycated hemoglobin levels was −0.3% (95% CI −0.46, −0.14). The numbers of patients with adverse events, serious adverse events or discontinued from study medication due to adverse events were similar in the omarigliptin and placebo groups.

**Conclusions:**

In this CV safety study of patients with T2DM and established CV disease, omarigliptin did not increase the risk of MACE or hHF and was generally well tolerated.

*Trial registration* ClinicalTrials.gov: NCT01703208. Registered 05 October 2012

**Electronic supplementary material:**

The online version of this article (doi:10.1186/s12933-017-0593-8) contains supplementary material, which is available to authorized users.

## Background

Omarigliptin is an oral dipeptidyl peptidase-4 (DPP-4) inhibitor for the treatment of type 2 diabetes mellitus (T2DM) with a half-life that allows once-weekly dosing [1]. Omarigliptin has previously been demonstrated to have efficacy comparable to that of the daily DPP-4 inhibitor sitagliptin [2, 3]. Omarigliptin has been marketed in Japan (as MARIZEV™) for the treatment of patients with T2DM since 2015.

When the omarigliptin Phase 3 development program was initiated in 2012, it was designed to support the approval of omarigliptin in multiple countries, including the United States (US). However, on April 8, 2016, the Sponsor, Merck & Co., Inc., Kenilworth, NJ, USA (known as MSD outside of the US and Canada), announced it would not proceed with submitting marketing applications for omarigliptin in the US or Europe. The decision was made for business reasons and was not related to safety or efficacy concerns about omarigliptin.

The omarigliptin Phase 3 program included a dedicated cardiovascular (CV) safety study (MK-3102-018; ClinicalTrials.gov NCT01703208), which was being conducted to meet the US Food and Drug Administration requirement to establish the CV safety of new therapies to treat T2DM to ensure that the therapy does not result in an unacceptable increase in CV risk. As part of this requirement, both pre-approval and post-approval assessments of CV safety were to be done, with the former met (based on a pooled analysis of adjudication-confirmed CV events from the Phase 3 program) and the latter requiring analysis of major adverse CV events (MACE), defined as the composite endpoint of first adjudication-confirmed event of CV death, nonfatal myocardial infarction (MI) or nonfatal stroke, based solely on the results of MK-3102-018. After the announcement by the Sponsor that approval of omarigliptin would not be pursued in the US or Europe, the CV safety study was no longer required to meet post-approval requirements and the study was terminated.

Three CV safety studies for the daily DPP-4 inhibitors saxagliptin (SAVOR-TIMI 53), alogliptin (EXAMINE) and sitagliptin (TECOS) have been completed and the results of these studies have significantly contributed to the scientific understanding of the CV safety of the DPP-4 inhibitor drug class [4–6]. These studies, which collectively randomized more than 36,500 patients (in a 1:1 ratio) to a daily DPP-4 inhibitor or placebo on a background of standard care, suggest that daily DPP-4 inhibitors are not associated with an increased risk for MACE, with the point estimates of the hazard ratios (HRs) for MACE approximately 1.0 in all three studies. An unexpected finding in SAVOR-TIMI 53 was that of an increased risk of hospitalization for HF (hHF) with saxagliptin (3.5% saxagliptin vs. 2.8% placebo; HR 1.27; 95% confidence interval [CI] 1.07, 1.51; p = 0.007) [6]. The EXAMINE study enrolled a post-acute coronary syndrome (ACS) population and did not show a statistically significant increased risk of hHF (3.9% alogliptin vs. 3.3% placebo; HR 1.19; 95% CI 0.90–1.58; p = 0.220) [7]. In TECOS, the incidence of hHF was similar in the sitagliptin and placebo groups (3.1% sitagliptin vs. 3.1% placebo; HR 1.00; 95% CI 0.83, 1.20; p = 0.98) [4]. These studies also served as sources of data to explore the overall safety of the DPP-4 inhibitor drug class, including pancreatic safety [8]. A meta-analysis suggested a small absolute increased risk for pancreatitis with DPP-4 inhibitor therapy (risk ratio 1.78 [95% CI 1.13, 2.81], p = 0.01), but not for pancreatic cancer (risk ratio 0.54 [95% CI 0.28, 1.04], p = 0.07) [8].

Herein we report the results of an analysis of CV endpoints and non-CV safety from the omarigliptin CV safety study (MK-3102-018), based on the data accrued up to the date of May 13, 2016, which was approximately 30 days after the announcement of study discontinuation. The rationale for a May 13, 2016 data cut-off date was to facilitate completion of the study and analysis of results. At the time of the data cut-off date, 403/4192 patients had not yet reached their final study visit, although most of these were discontinued shortly afterwards.

## Methods

### Patients

**Fig. 1:**
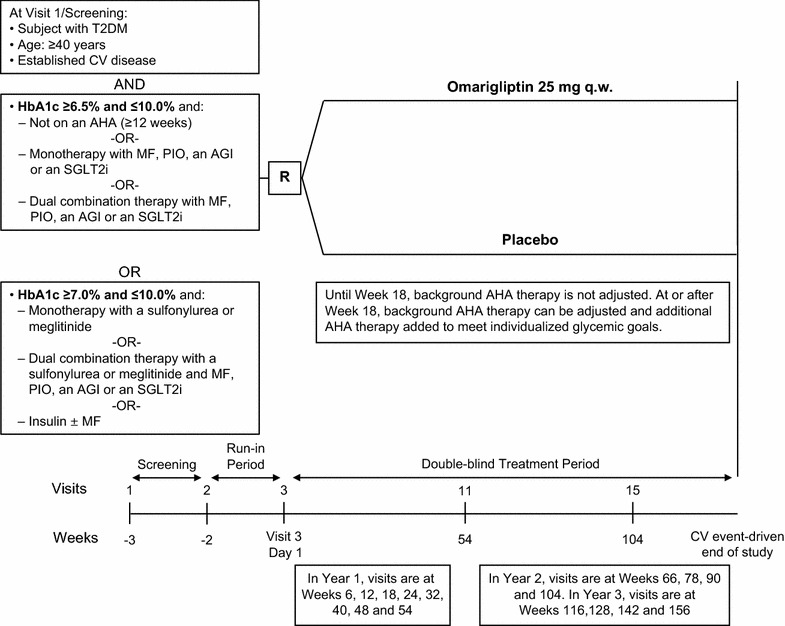
Study design; *T2DM* type 2 diabetes, *CV* cardiovascular, *AHA* antihyperglycemic agent, *MF* metformin, *PIO* pioglitazone, *AGI* alpha-glucosidase inhibitor, *SGLT2i* sodium–glucose co-transporter 2 inhibitor, *SU* sulfonylurea, *q.w.* once weekly, *R* randomization

Eligible patients were at least 40 years old with a history of T2DM and established CV disease. Patients were required to be on one of the following diabetes treatment regimens that was stable for at least 12 weeks (except for pioglitazone which was required to be stable for at least 16 weeks) and be within the glycated hemoglobin (HbA1c) range associated with the treatment regimen (Fig. 1). Patients on diet and exercise alone (not on an AHA for ≥12 weeks) or on monotherapy or dual combination therapy with metformin (MF), pioglitazone (PIO), an alpha-glucosidase inhibitor (AGI) or an SGLT2 inhibitor (SGLT2i) were required to have an HbA1c of ≥6.5 and ≤10.0% (≥48 and ≤86 mmol/mol). Patients on a sulfonylurea or meglitinide either as monotherapy or as part of dual combination therapy with MF, PIO, AGI or an SGLT2i were required to have an HbA1c ≥7.0 and ≤10.0% (≥53 mmol/mol and ≤86 mmol). Patients could also be enrolled if they were on a stable insulin regimen (±metformin) with an HbA1c ≥7.0 and ≤10.0% (≥53 mmol/mol and ≤86 mmol). Insulin regimens that were allowed included basal insulin (e.g., insulin glargine, insulin detemir, NPH insulin, degludec), prandial insulin (e.g., regular, aspart, lispro, glulisine), basal/prandial insulin regimen consisting of multiple dose insulin injections of basal and prandial insulin or the use of pre-mixed insulin (e.g., Novolog 70/30^®^, Novolin 70/30^®^, Humalog 75/25^®^, or Humulin 70/30^®^). A stable insulin regimen was defined as no change in the insulin regimen [i.e. type(s) of insulin] and ≤10% change in the total daily dose of insulin. Prior to randomization, patients were required to have a site fasting-fingerstick glucose (FFSG) >126 mg/dL (7.0 mmol/L) and <260 mg/dL (14.4 mmol/L).

The criteria for having established CV disease included the presence of one of the following: (1) coronary artery disease defined as having a history of MI, surgical or percutaneous (balloon and/or stent) coronary revascularization procedure, or coronary angiography showing at least 1 stenosis ≥50% in a major epicardial artery or branch vessel; (2) ischemic cerebrovascular disease defined as having a history of ischemic stroke (strokes not known to be hemorrhagic will be allowed as part of this criterion); (3) carotid arterial disease defined as a ≥50% stenosis documented by carotid ultrasound, magnetic resonance imaging, or angiography, with or without symptoms of neurologic deficit; or (4) atherosclerotic peripheral arterial disease, as documented by objective evidence such as amputation due to vascular disease, current symptoms of intermittent claudication confirmed by an ankle-brachial pressure index of <0.9 or a toe-brachial pressure index <0.7 or history of surgical or percutaneous revascularization procedure.

Patients were excluded if they had acute coronary syndrome (e.g., MI or unstable angina), a coronary artery intervention (e.g., CABG or PTCA), a stroke or transient ischemic neurological disorder or worsening signs or symptoms of coronary heart disease or congestive heart failure within the past 3 months; type 1 diabetes, a history of ketoacidosis, or C-peptide <0.7 ng/mL; estimated glomerular filtration rate <40 mL/min/1.73 m^2^; active liver disease or an aspartate aminotransferase (AST) or alanine aminotransferase (ALT) level >2× the upper limit of normal (ULN); thyroid stimulating hormone outside the central laboratory normal range; triglycerides >600 mg/dL (6.78 mmol/L); hemoglobin <12 g/dL (120 g/L) (males) and <11 g/dL (110 g/L) (females); history of malignancy or hematological disorders; or previously treated with omarigliptin.

### Study design

This multicenter, double-blind, randomized, placebo-controlled, parallel group, Phase 3 study (Fig. 1) was conducted in 559 centers: 10 in Argentina, 7 in Australia, 5 in Austria, 10 in Belgium, 16 in Brazil, 6 in Bulgaria, 7 in Canada, 4 in Chile, 6 in Colombia, 6 in Croatia, 11 in the Czech Republic, 7 in Denmark, 2 in Finland, 5 in France, 15 in Georgia, 24 in Germany, 8 in Hong Kong, 8 in Hungary, 11 in Israel, 14 in Italy, 4 in Lebanon, 5 in Lithuania, 9 in Malaysia, 4 in Mexico, 5 in the Netherlands, 7 in New Zealand, 12 in the Philippines, 14 in Poland, 11 in the Republic of Korea, 16 in Romania, 12 in the Russian Federation, 5 in Serbia, 11 in Slovakia, 20 in South Africa, 15 in Spain, 9 in Sweden, 6 in the province of Taiwan, 16 in the Ukraine, 16 in the United Kingdom and 180 in the United States. Patients were randomly assigned in a 1:1 ratio to omarigliptin 25 mg q.w. or placebo using an interactive voice-response system.

After Week 18, patients were to have their background AHA regimen adjusted as deemed necessary by the study investigator to achieve an appropriate individualized glycemic goal in line with local, national or international guidelines. Whenever possible, the patients were followed until study closeout, regardless of whether they continued to receive blinded study medication. The study was designed and funded by the Sponsor and conducted in conjunction with PAREXEL International Corp., Waltham, MA.

The study (Omarigliptin Protocol 018; ClinicalTrials.gov: NCT01703208) was conducted in accordance with the principles of Good Clinical Practice and was approved by the appropriate institutional review boards and regulatory agencies. All patients enrolled in the study provided written informed consent.

### Study evaluations

Safety assessment included collection of adverse events, physical examination, vital signs, standard laboratory blood chemistry (e.g., liver and renal safety tests), lipid panel, hematology, urinalysis and electrocardiogram. In addition, amylase and lipase were measured per regulatory agency request. A standard questionnaire was provided to patients to collect hypoglycemia information.

An external Data Monitoring Committee reviewed unblinded interim data from this study. External Clinical Adjudication Committees (CACs) evaluated potential CV events, cases of pancreatitis and prespecified hypersensitivity reactions in a blinded manner. The CACs were not charged with assessing causality to study medication. The adjudication of hypersensitivity was at the request of regulatory authorities and was not due to any signal observed with omarigliptin. Hypersensitivity events prespecified for adjudication were anaphylactic reaction, angioedema, asthma–bronchospasm, erythema multiforme, Stevens–Johnson syndrome, toxic epidermal necrolysis, and drug rash with eosinophilia and systemic symptoms.

### Endpoints

CV endpoints analyzed included (1) time to first event of MACE (confirmed CV-related death, nonfatal MI, nonfatal stroke); (2) time to confirmed CV-related death; (3) time to first event of confirmed MI (fatal and nonfatal); (4) time to first event of stroke (fatal and nonfatal); (5) time to all-cause mortality; (6) time to first event of confirmed hHF; and (7) time to the composite of first confirmed event hHF or CV death. Change from baseline over time in HbA1c and non-CV safety, including hypoglycemia, was analyzed. Criteria for the adjudication of CV endpoints followed guidelines of the Clinical Data Interchange Standards Consortium for standardized definition of CV [9] and stroke endpoints in clinical trials and the Third Universal Definition of Myocardial Infarction [10], as excerpted in Additional file 1.

### Statistical analyses

The number of patients randomized was based on the primary hypothesis of the study, which was non-inferiority of omarigliptin compared with placebo for the primary composite MACE endpoint. Non-inferiority was to be declared if the upper bound of the two-sided 95% confidence interval (CI) of the HR for MACE was <1.30 in the intention-to-treat (ITT) population and was to include all confirmed events that occurred up to 365 days after last dose of blinded study medication. A total of 632 patients having a primary endpoint event would provide 90% power for the test of non-inferiority, assuming a true HR of 1.00. It was estimated that the study would take approximately 8 years from initiation to completion, assuming accrual of primary endpoints at 3.0% per year and rates of enrollment and patient dropout.

Due to the early termination of the study and the attendant decrease in power to adequately test the primary hypothesis of non-inferiority, no formal statistical testing of the non-inferiority hypothesis was conducted. The population analyzed was the ITT population that included all randomized patients who took at least 1 dose of blinded study medication and all CV endpoint data, regardless of the time since a patient may have discontinued blinded study medication.

The analyses presented herein encompass the time period from first-patient-first-visit (October 16, 2012) to the final data cut-off date (May 13, 2016). The data cut-off date was approximately 30 days after investigator notification of study discontinuation, which followed the Sponsor’s announcement of its intent not to seek marketing authorization for omarigliptin in the US and Europe. At the time of the data cut-off date, 90.4% of randomized patients had been discontinued from the study. CV events that occurred after the data cut-off date in the remaining 9.6% of patients (n = 403; 209 in the omarigliptin group and 194 in the placebo group) who were ongoing in the study at the time of the data cut-off date were not adjudicated and are not included in the analyses presented herein. Nearly all of the ongoing patients were discontinued from blinded study medication shortly afterwards; however, in Brazil, based on local regulatory procedures, 75 patients remained on blinded study medication, with the last patient visit occurring on March 23, 2017.

The ITT population was used for the assessment of CV endpoints. The Cox proportional-hazards model was used to calculate the HR and two-sided 95% CI. The Kaplan–Meier method was used to estimate the time-to-first event in the two treatment groups. The Full Analysis Set, consisting of all randomized patients who received at least 1 dose of study medication and had a baseline or a post-randomization measurement, was used for analyzing the HbA1c endpoint. No subgroup analyses were performed. Safety and tolerability were assessed in the All-Patients-as-Treated (APaT) population, which included all randomized patients who received at least 1 dose of study medication, by clinical review of all relevant parameters, including adverse events, laboratory tests and vital signs.

The primary analysis of safety was assessed in the time frame consisting of the Treatment Period + 21 days. Adverse events of symptomatic hypoglycemia (episodes with clinical symptoms attributed to hypoglycemia, without regard to glucose level) were prespecified as events of interest and p values and 95% CI for between-treatment group comparisons were calculated. Confirmed events of pancreatitis and prespecified hypersensitivity adverse events were summarized regardless of time after discontinuation from study medication. Patients who discontinued blinded study medication and who did not withdraw consent were followed by telephone contact until study end to ascertain any serious adverse events that occurred after the discontinuation of blinded study medication. A second approach to safety analyses, that applied only to serious adverse events, included all safety-related data after the first dose of blinded study medication and was referred to as Treatment Period + all follow-up days.

## Results

### Patient disposition and characteristics

Patient disposition is summarized in Table 1. Eight patients randomized to the omarigliptin group and two randomized to the placebo group did not take any study medication and were not included in any safety or efficacy analyses. Demographic and anthropometric characteristics of randomized patients were similar between the two treatment groups (Table 2). Approximately 70% of patients were male and 30% female; the mean age was 63.6 years. Racial percentages were 81.3% White, 10.8% Asian, 3.8% Black, and 12.7% were of Hispanic or Latino ethnicity. The mean BMI was 31.3 kg/m^2^. Twenty-one percent of patients were from North America, 55.6% were from Europe, 6.8% were from Latin America, 9.1% were from Asia and 7.6% were from other countries.

**Table 1:** Disposition of patients

Study medication disposition, n (%)	Omarigliptin 25 mg *N* = 2100	Placebo *N* = 2102
Did not take study medication	8 (0.4)	2 (0.1)
Discontinued study medication	2092 (99.6)	2100 (99.9)
Adverse event	88 (4.2)	74 (3.5)
Death	48 (2.3)	37 (1.8)
Lack of efficacy	9 (0.4)	14 (0.7)
Lost to follow-up	44 (2.1)	54 (2.6)
Non-compliance with study drug	10 (0.5)	6 (0.3)
Physician decision	25 (1.2)	33 (1.6)
Progressive disease	2 (0.1)	1 (0.0)
Protocol violation	7 (0.3)	14 (0.7)
Study terminated by sponsor	1615 (76.9)	1602 (76.2)
Withdrawal by patient	197 (9.4)	213 (10.1)
Other	47 (2.2)	52 (2.5)

**Table 2:** Baseline characteristics of study patients

	Omarigliptin *N* = 2100	Placebo *N* = 2102
Age, years	63.7 ± 8.5	63.6 ± 8.5
Male, *n* (%)	1461 (69.6)	1487 (70.7)
Race, *n* (%)		
White	1707 (81.3)	1709 (81.3)
Asian	234 (11.1)	221 (10.5)
Black	73 (3.5)	87 (4.1)
Multi-racial	67 (3.2)	67 (3.2)
American Indian/Alaska Native	13 (0.6)	12 (0.6)
Native Hawaiian or Other Pacific Islander	6 (0.3)	6 (0.3)
Ethnicity, *n* (%)		
Not Hispanic or Latino	1813 (86.3)	1814 (86.3)
Hispanic or Latino	267 (12.7)	266 (12.7)
Not reported	13 (0.6)	10 (0.5)
Unknown	7 (0.3)	12 (0.6)
Body weight, kg	89.0 ± 18.5	89.6 ± 18.8
BMI, kg/m^2^	31.2 ± 5.5	31.4 ± 5.6
Geographic region		
North America	431 (20.5)	450 (21.4)
Europe	1166 (55.5)	1169 (55.6)
Latin America	140 (6.7)	144 (6.9)
Asia	201 (9.6)	180 (8.6)
Other^a^	162 (7.7)	159 (7.6)
HbA1c, %	8.0 ± 0.9	8.0 ± 0.9
Duration of diabetes, years	12.0 ± 7.6	12.1 ± 8.0
eGFR (mL/min/1.73 m^2^)	85.7 ± 24.5	86.6 ± 25.5
History of congestive heart failure	341 (16.2)	300 (14.3)
History of hypertension	1998 (95.1)	2010 (95.6)
Cigarette smoking status		
Current	301 (14.3)	305 (14.5)
Former	825 (39.3)	815 (38.8)
Never	974 (46.4)	981 (46.7)
Unknown	0 (0.0)	1 (0.0)
Prior AHA therapy		
Insulin	769 (36.6)	699 (33.3)
Metformin	1646 (78.4)	1606 (76.4)
Sulfonylurea	817 (38.9)	826 (39.3)
SGLT2 inhibitor	4 (0.2)	4 (0.2)
Thiazolidinedione	24 (1.1)	21 (1.0)
Other	55 (2.6)	55 (2.6)
None	71 (3.4)	84 (4.0)

Values are mean ± SD or n (%)

*BMI* body mass index, *eGFR* estimated glomerular filtration rate, *AHA* anti-hyperglycemic agent, *SGLT2* sodium–glucose linked transporter 2

^a^For geographic region, other includes Australia, Israel, Lebanon, New Zealand and South Africa

Baseline disease state characteristics are presented in Table 2. The mean baseline HbA1c was 8.0% and mean duration of T2DM was 12.1 years. The majority of patients (88.3%) had an estimated glomerular filtration rate (eGFR) ≥60 mL/min/1.73 m^2^ and 11.3% had an eGFR ≥30 and <60 mL/min/1.73 m^2^. The majority had a history of hypertension (95.4%) and 15.3% had a history of heart failure (16.2% in the omarigliptin group and 14.3% in the placebo group). The most commonly used prior oral AHA medication was metformin (77.4%), followed by sulfonylurea (39.1%) and insulin (34.9%).

The median duration of exposure to study medication was similar in the two treatment groups: 90.0 weeks (range 1.0–172.0) in the omarigliptin group and 89.0 weeks (range 1.0–168.0) in the placebo group. The median duration of treatment plus post-treatment follow-up was similar in the two treatment groups: 96.1 weeks (range 1.1–178.6) in the omarigliptin group and 95.6 weeks (range 1.3–176.0) in the placebo group. Mean compliance with study medication (omarigliptin or matching placebo) was similar for the two treatment groups (97.8 and 97.9% for omarigliptin and placebo, respectively).

### CV endpoints

Table 3 summarizes the results of the CV endpoints analyzed. There was no significant between-group difference in the composite MACE endpoint, which occurred in 114/2092 patients in the omarigliptin group (5.45%) and 114/2100 patients in the placebo group (5.43%).

**Table 3:** Cardiovascular endpoints in the ITT population; Treatment Period + all follow-up days

Endpoint	Number of events (%)		Rate/100 patient-years^a^		Hazard ratio of omarigliptin vs. placebo (95% CI)^b^
	Omarigliptin *N* = 2092	Placebo *N* = 2100	Omarigliptin	Placebo	
Cardiovascular death, nonfatal myocardial infarction or nonfatal stroke	114 (5.45)	114 (5.43)	2.96	2.97	1.00 (0.77, 1.29)
Cardiovascular-related death	37 (1.77)	35 (1.67)	0.94	0.89	1.06 (0.66, 1.68)
Fatal and nonfatal myocardial infarction	52 (2.49)	60 (2.86)	1.34	1.55	0.87 (0.60, 1.26)
Fatal and nonfatal stroke	32 (1.53)	34 (1.62)	0.82	0.88	0.94 (0.58, 1.52)
All-cause mortality	64 (3.06)	50 (2.38)	1.63	1.28	1.28 (0.88, 1.85)
Hospitalization for heart failure	20 (0.96)	33 (1.57)	0.51	0.85	0.60 (0.35, 1.05)
Hospitalization for heart failure or CV death	55 (2.63)	64 (3.05)	1.41	1.65	0.86 (0.60, 1.23)

^a^Patient-years is calculated as the sum of all patients follow-up time to event. For patients without an event, the time to event is the last follow-up time as defined for ITT population in the statistical analysis plan

^b^Based on the proportional hazards model that includes treatment as an explanatory factor

**Fig. 2:**
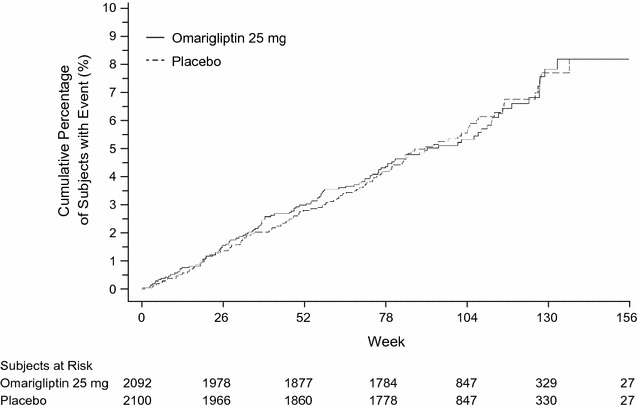
Kaplan–Meier plot of time to first major adverse cardiac event in the intention-to-treat population

Figure 2 presents the Kaplan–Meier plot of time to first MACE event. There was no notable treatment difference in event rates over time through Week 156.

**Fig. 3:**
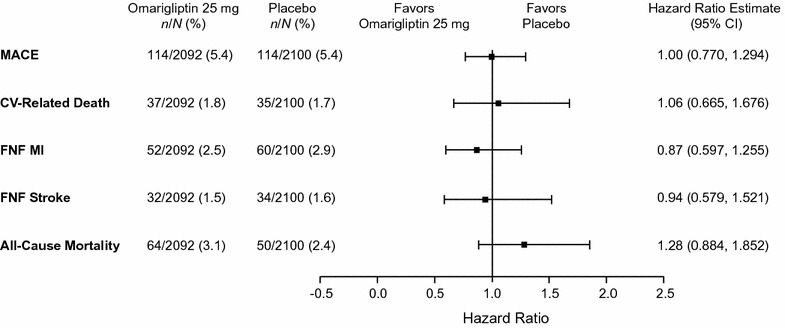
Forest plot of the hazard ratios for major adverse cardiac events (MACE) and other cardiovascular (CV) endpoints [CV-related death, fatal and nonfatal (FNF) MI, FNF stroke, and all-cause mortality]

The rate of confirmed MACE per 100 patient-years was 2.96 for omarigliptin and 2.97 for placebo (HR, 1.00; 95% CI, 0.77–1.29). The rate of confirmed CV-related death per 100 patient-years was 0.94 for omarigliptin and 0.89 for placebo, with an HR (95% CI) of 1.06 (0.66, 1.68). The rate of confirmed fatal or non-fatal MI per 100 patient-years was 1.34 for omarigliptin and 1.55 for placebo, with an HR (95% CI) of 0.87 (0.60, 1.26). The rate of confirmed fatal or non-fatal stroke per 100 patient-years was 0.82 for omarigliptin and 0.88 for placebo, with an HR (95% CI) of 0.94 (0.58, 1.52). The rate of all-cause mortality per 100 patient-years was 1.63 for omarigliptin and 1.28 for placebo, with an HR (95% CI) of 1.28 (0.88, 1.85). There was no specific pattern of non-CV deaths. Figure 3 presents a forest plot of MACE and other CV endpoints (CV-related death, fatal and nonfatal MI, fatal and nonfatal stroke and all-cause mortality).

There was no significant difference in the rate of hHF or the rate of the composite of hHF and CV death. The rate of first hHF per 100 patient-years was 0.51 for omarigliptin and 0.85 for placebo, with an HR (95% CI) of 0.60 (0.35, 1.05). The rate of the composite endpoint of first hHF or CV death per 100 patient-years was 1.41 for omarigliptin and 1.65 for placebo, with an HR (95% CI) of 0.86 (0.60, 1.23).

### Change from baseline in HbA1c

**Fig. 4:**
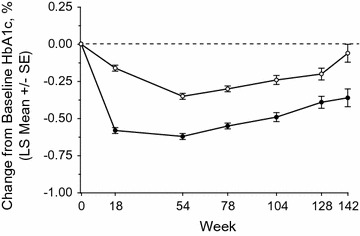
Changes from baseline in HbA1c over time; *filled circle* omarigliptin 25 mg q.w., *open circle* placebo

The changes from baseline in HbA1c over time (up to Week 142) are shown in Fig. 4. At Week 18, prior to the adjustment of background medication to meet individualized patient goals (see above), from a mean baseline HbA1c of 8.0%, the LS mean change from baseline (95% CI) in HbA1c was −0.58% (−0.62, −0.55) in the omarigliptin group and −0.16% (−0.19, −0.12) in the placebo group, with a between treatment difference of −0.43% (−0.48, −0.37). The profile of the change in HbA1c over time indicates that the between-group difference persisted throughout the treatment period. At Week 142, the change from baseline (95% CI) was −0.36% (−0.47, −0.25) in the omarigliptin group and −0.06% (−0.17, 0.05) in the placebo group, with a between treatment difference of −0.30% (−0.46, −0.14). Over time, an increase in HbA1c was observed in both treatment groups.

### Non-cardiovascular safety

In the following, the term “higher” indicates that the 95% CI for the between-group difference in the incidences of adverse events excluded 0. Adverse event summary measures were similar in the omarigliptin and placebo group, with the exception of drug-related adverse events, which was higher in the omarigliptin group compared with the placebo group (Table 4). The between-group difference in the overall incidence of drug-related adverse events was primarily due to differences in the adverse event of hypoglycemia.

**Table 4:** Adverse event (AE) summary measures in the APaT population; Treatment Period + 21 days

Patients, *n* (%)	Omarigliptin *N* = 2092	Placebo *N* = 2100	Difference^a^
With one or more			
AEs	1605 (77.2)	1583 (75.4)	1.8 (−0.8, 4.4)
Drug-related^b^ AEs	268 (12.8)	215 (10.2)	2.6 (0.6, 4.5)
Serious AEs	476 (22.8)	467 (22.2)	0.5 (−2.0, 3.0)
Serious drug-related^b^ AEs	8 (0.4)	6 (0.3)	0.1 (−0.3, 0.5)
Who died	39 (1.9)	30 (1.4)	0.4 (−0.3, 1.2)
Who discontinued due to			
An AE	77 (3.7)	68 (3.2)	0.4 (−0.7, 1.6)
A drug-related^b^ AE	21 (1.0)	18 (0.9)	0.1 (−0.5, 0.8)
A serious AE	37 (1.8)	28 (1.3)	0.4 (−0.3, 1.2)
A serious drug-related^b^ AE	3 (0.1)	1 (0.0)	0.1

^a^Difference in % vs. placebo; estimate (95% CI) was computed only for AE summary endpoints with at least four patients having events in one or more treatment groups

^b^Assessed by the investigator as related to study drug

The number of patients with adverse events by MedDRA system organ class (SOC) was similar between the omarigliptin and placebo groups, with the exception of skin and subcutaneous tissue disorders SOC, where the incidence was higher in the omarigliptin group compared with the placebo group (7.9% [166/2092 patients] versus 6.0% [126/2100 patients]) due to a variety of specific adverse events. The majority of cutaneous adverse events in the omarigliptin group were non-serious; however 2 (1 eczema and 1 dermatosis) were serious, but were not considered by the investigator to be related to study medication and did not lead to the discontinuation of study medication.

Among the specific adverse events, the incidence of the adverse event of AST increased was higher in the omarigliptin group compared with the placebo group (Table 5). The between-group difference in adverse event of ALT increased was less notable and there were no notable between-group differences in the incidences of exceeding the predefined limits of change (PDLC) for ALT or AST of ≥3× ULN or ≥5× ULN. Including supplemental laboratory data (measurements entered in the database from sources other than the central laboratory; primarily drawn during hospitalization), there were 16 patients in the omarigliptin group and 17 patients in the placebo group with ALT ≥5× ULN; all of the patients in the omarigliptin group had a plausible alternative explanation (e.g., acute cholecystitis, alcohol abuse, metastatic cancer) and/or resolved on study medication. There were no cases that met the combined criteria for a Hy’s law case (i.e., ALT or AST ≥3× ULN with elevations in bilirubin ≥2× ULN and alkaline phosphatase <2× ULN without alternative explanation) that would suggest the potential for drug-induced liver injury [11]. There were no between-group differences in mean change from baseline over time in ALT or AST.

**Table 5:** Incidences of adverse events related to alanine aminotransferase (ALT) and aspartate aminotransferase (AST) and predefined limits of change for those liver tests in the APaT population, Treatment Period + 21 days; and change from baseline in ALT and AST at Week 54

Adverse event, *n* (%)	Omarigliptin *N* = 2092	Placebo *N* = 2100
ALT increased	24 (1.1)	16 (0.8)
AST increased	16 (0.8)	4 (0.2)
Predefined limit of change		
ALT ≥3× ULN	35 (1.7)	34 (1.6)
AST ≥3× ULN	19 (0.9)	16 (0.8)
ALT ≥5× ULN	14 (0.7)	12 (0.6)
AST ≥5× ULN	10 (0.5)	6 (0.3)
ALT ≥5× ULN^a^	16 (0.7)	17 (0.8)
Mean change from baseline (SD) at Week 54^b^ (IU/L)		
ALT	−0.6 (15.2)	−0.6 (12.5)
AST	0.2 (14.5)	−0.4 (9.7)

Central laboratory normal range: ALT 10–40 IU/L (male) and 10–33 IU/L (female); AST 10–43 IU/L (male) and 10–36 IU/L (female)

^a^Including supplemental laboratory data

^b^Mean changes based on *n* = 1801 in the omarigliptin group and *n* = 1756 in the placebo group

Table 6 summarizes the adverse events of hypoglycemia. The incidence of symptomatic hypoglycemia was higher in the omarigliptin group (22.6% [472/2092 patients]) compared with the placebo group (19.7% [414/2100 patients]); p = 0.024. The incidence of severe hypoglycemia requiring medical assistance was 4.2% (88/2092 patients) in the omarigliptin group compared to 3.3% in (70/2100 patients) in the placebo group.

**Table 6:** Analysis of adverse events (AEs) of hypoglycemia in the APaT population Treatment Period + 21 days

Patients, *n* (%)	Omarigliptin *N* = 2092	Placebo *N* = 2100	Difference^a^
With one or more AEs of hypoglycemia	513 (24.5)	454 (21.6)	2.9 (0.4, 5.5)
Symptomatic^b^	472 (22.6)	414 (19.7)	2.8^f^ (0.4, 5.3)
Documented symptomatic^c^	447 (21.4)	387 (18.4)	2.9 (0.5, 5.4)
Severe^d^	88 (4.2)	70 (3.3)	0.9 (−0.3, 2.0)
Requiring non-medical assistance	82 (3.9)	58 (2.8)	1.2 (0.1, 2.3)
Requiring medical assistance	11 (0.5)	19 (0.9)	−0.4 (−0.9, 0.1)
Asymptomatic^e^	126 (6.0)	123 (5.9)	0.2 (−1.3, 1.6)

^a^Difference in % vs. placebo

^b^Symptomatic hypoglycemia: episode with clinical symptoms attributed to hypoglycemia, without regard to glucose level

^c^Documented symptomatic: episode with clinical symptoms attributed to hypoglycemia with a documented glucose levels of ≤3.9 mmol/L (≤70 mg/dL)

^d^Severe hypoglycemia: episode that required assistance, either medical or non-medical. Episodes with a markedly depressed level of consciousness, a loss of consciousness, or seizure were classified as having required medical assistance, whether or not medical assistance was obtained

^e^Asymptomatic hypoglycemia: finger stick glucose values ≤3.9 mmol/L (70 mg/dL) without symptoms

^f^p = 0.024

Table 7 summarizes the events of adjudication-confirmed acute and chronic pancreatitis and adjudication-confirmed prespecified hypersensitivity reactions. Confirmed acute pancreatitis events were uncommon overall and none of the cases in either treatment group were fatal. One patient in the placebo group had confirmed chronic pancreatitis (no cases occurred in the omarigliptin group).

**Table 7:** Incidences of adjudication-confirmed cases of pancreatitis and prespecified hypersensitivity adverse events in the APaT population; Treatment Period + all follow-up days

Patients, n (%)	Omarigliptin *N* = 2092	Placebo *N* = 2100
With pancreatitis		
Acute	6 (0.3)	3 (0.1)
Chronic	0 (0.0)	1 (<0.1)
With hypersensitivity		
Angioedema	4 (0.2)	1^a^ (0.1)
Asthma–bronchospasm	2 (0.1)	2 (0.1)
Anaphylaxis	0 (0.0)	1 (<0.1)

^a^One patient in the placebo group was reported to have experienced two cases of angioedema

Four patients in the omarigliptin group had a confirmed case of angioedema; all were nonserious and one case occurred in the post-treatment period (i.e., after the 21-day follow-up after discontinuation of study medication). One patient in the placebo group had two confirmed cases of angioedema; both of these cases were serious adverse events. Two patients in the omarigliptin group with a history of asthma had events of confirmed asthma–bronchospasm; one was reported as non-serious and the other as a serious adverse event. Two patients in the placebo group had confirmed cases of asthma–bronchospasm; both were reported as non-serious. One patient in the placebo group had a confirmed case of anaphylactic reaction.

Investigator reported pancreatic cancer occurred in five patients in the omarigliptin group (3 cases of pancreatic carcinoma and 2 cases of pancreatic cancer metastatic) and one patient in the placebo group (1 case of pancreatic carcinoma).

There were no clinically meaningful changes from baseline or in analysis of PDLC in laboratory safety measures. Small changes from baseline in mean serum amylase and lipase values were observed in the omarigliptin group by Week 6 and continued through Week 156 (Table 8). All mean serum amylase values over time were within the normal laboratory range (35–121 U/L) throughout the treatment period in both treatment groups. The mean serum lipase value was within the normal laboratory range (13–60 U/L) in both treatment groups throughout the treatment period except for the omarigliptin group at Week 104, when the mean value (72.3 IU/L ± 437.2) was slightly greater than the upper limit of the normal range.

**Table 8:** Mean (IU/L) ± standard deviation serum amylase and lipase values through Week 156

Week	Serum amylase		Serum lipase	
	Omarigliptin *N* = 2092	Placebo *N* = 2100	Omarigliptin *N* = 2092	Placebo *N* = 2100
0	66.5 ± 33.3 *n* = 1465	67.5 ± 39.2 *n* = 1457	45.1 ± 35.4 *n* = 1465	45.5 ± 53.5 *n* = 1457
6	71.3 ± 34.0 *n* = 1353	66.7 ± 35.1 *n* = 1338	53.4 ± 45.6 *n* = 1353	45.1 ± 50.1 *n* = 1337
12	72.9 ± 35.8 *n* = 1329	68.1 ± 42.7 *n* = 1301	56.2 ± 49.9 *n* = 1329	46.4 ± 41.7 *n* = 1300
18	71.6 ± 35.3 *n* = 1314	67.4 ± 35.2 *n* = 1281	52.1 ± 42.2 *n* = 1313	45.1 ± 39.0 *n* = 1280
24	72.0 ± 34.8 *n* = 1305	67.6 ± 33.4 *n* = 1296	55.6 ± 52.5 *n* = 1305	45.5 ± 35.0 *n* = 1295
32	71.3 ± 33.7 *n* = 1308	68.1 ± 34.8 *n* = 1292	51.4 ± 33.2 *n* = 1308	46.4 ± 42.9 *n* = 1292
40	70.9 ± 35.4 *n* = 1289	67.1 ± 33.8 *n* = 1261	51.3 ± 37.5 *n* = 1289	43.7 ± 29.3 *n* = 1261
48	71.6 ± 34.6 *n* = 1282	67.5 ± 35.9 *n* = 1235	54.2 ± 54.2 *n* = 1280	44.6 ± 36.1 *n* = 1236
54	74.1 ± 52.6 *n* = 1292	68.2 ± 33.5 *n* = 1253	56.3 ± 185.2 *n* = 1291	44.1 ± 37.9 *n* = 1253
66	72.8 ± 35.8 *n* = 1263	68.8 ± 42.4 *n* = 1229	54.3 ± 56.3 *n* = 1260	44.5 ± 37.1 *n* = 1229
78	75.1 ± 57.9 *n* = 1339	67.5 ± 33.9 *n* = 1297	53.4 ± 50.2 *n* = 1339	47.2 ± 61.9 *n* = 1294
90	72.1 ± 38.0 *n* = 836	69.1 ± 38.8 *n* = 808	57.4 ± 74.0 *n* = 835	46.1 ± 40.5 *n* = 808
104	74.8 ± 85.9 *n* = 612	68.3 ± 42.1 *n* = 568	72.3 ± 437.2 *n* = 611	45.1 ± 32.6 *n* = 568
116	74.7 ± 38.2 *n* = 376	65.5 ± 30.8 *n* = 351	59.0 ± 84.0 *n* = 376	46.6 ± 54.2 *n* = 351
128	75.7 ± 36.2 *n* = 213	64.1 ± 32.7 *n* = 223	58.4 ± 52.6 *n* = 213	42.9 ± 24.5 *n* = 223
142	70.7 ± 30.8 *n* = 69	71.7 ± 54.2 *n* = 55	52.8 ± 31.2 *n* = 69	44.0 ± 25.9 *n* = 55
156	70.0 ± N/A *n* = 1	41.0 ± N/A *n* = 1	31.0 ± N/A *n* = 1	9.0 ± N/A *n* = 1

Measurement of amylase and lipase was added to the protocol after study initiation at the request of several European countries; therefore not all patients had baseline values

Central laboratory normal range: serum amylase = 35–121 U/L; serum lipase = 13–60 U/L

There were no clinically meaningful between-group changes from baseline in eGFR at any timepoint. At Week 142, the LS mean change from baseline (SE) in eGFR calculated by the MDRD equation (mL/min/1.73 m^2^) was −0.97 (1.05) in the omarigliptin group and 1.45 (1.06) in the placebo group; LS mean difference (95% CI) was −2.43 (−5.36, 0.51).

There were no clinically meaningful changes from baseline over time in heart rate, blood pressure, or ECG intervals (including QTc) in either treatment group. At Week 142, the LS mean changes (SE) from baseline in body weight were −0.33 kg (0.21) in the omarigliptin group and −0.25 kg (0.22) in the placebo group.

## Discussion

The results of this study suggest that treatment of patients with T2DM and established CV disease with once-weekly omarigliptin is not associated with an increased risk of MACE. These results are consistent with the three completed CV outcome studies with daily DPP-4 inhibitors [4–6]. The 1.5 years median follow-up of this study due to its early termination is shorter than that of the completed CV safety studies with similar designs and patient populations for the DPP-4 inhibitors saxagliptin (SAVOR-TIMI 53) [6] and sitagliptin (TECOS) [4], which had median follow-ups of 2.1 and 3.0 years, respectively, but was similar to the median follow-up in EXAMINE [5], although the latter study enrolled a different (post-ACS) population.

In this study, analysis was performed on approximately 36% (i.e., a total of 228 confirmed CV events) of the 632 confirmed CV events projected to be needed to provide >90% power to demonstrate non-inferiority. As is the case with any early-terminated safety study, the effects of treatment on endpoints observed during the period prior to study termination might differ from those observed at later timepoints. Nonetheless, in the present study a high degree of consistency in the rate of CV events was observed between treatment groups up until the time of study termination, and the observed profile of accrued CV events over time was consistent with that of previously completed CV safety studies.

In this study, there was no increase observed in the risk of hHF, although the number of confirmed first events of hHF was modest. Nonetheless, the results of the study are consistent with the results of TECOS, which did not show an increased risk of hHF with sitagliptin [4].

The analysis of the change from baseline in HbA1c indicates omarigliptin provided a clinically meaningful reduction in HbA1c throughout the treatment period. In the first 18 weeks of the study, during which time the effects of omarigliptin therapy on HbA1c would be expected to achieve near-maximum, investigators were instructed not to adjust background AHAs unless required to avoid hypoglycemia or excessive hyperglycemia. As anticipated, the largest between-group difference in HbA1c was observed at Week 18 (−0.43%). Subsequently, despite the fact that investigators were encouraged to adjust AHAs after Week 18 to meet individualized patient goals, a between-group difference in HbA1c persisted throughout the treatment period.

The present study provided a sizable database for an analysis of non-CV safety. The analysis of hypoglycemia suggests that omarigliptin has a favorable profile compared with placebo on a background of a variety of diabetes medications, including insulin. The incidence of symptomatic hypoglycemia in the omarigliptin group compared with the placebo group (22.6% vs. 19.7%) is consistent with previous observations of daily DPP-4 inhibitors, which can be associated with an increased risk of hypoglycemia when co-administered with drugs that are associated with hypoglycemia, such as insulin and sulfonylureas [12–14]. Approximately 39% of patients in both treatment groups were treated with insulin and 42% were treated with a sulfonylurea.

The observed imbalance in the incidence of the adverse event of AST increased prompted a detailed review of other liver test-related adverse events and a review of PDLC and mean changes from baseline in liver tests. No imbalances were observed in the PDLC for liver tests, which is an objective assessment of those measures, as opposed to adverse events, which are a more subjective measure due to investigator discretion about reporting an abnormal laboratory value as an adverse event. No cases meeting the definition of Hy’s law [11] were identified. The conclusion from this review is that omarigliptin does not pose a risk for clinically important liver events.

In past years, the topic of pancreatic safety of incretin therapies has been raised [15]. The results of this study indicate that acute (6 omarigliptin versus 3 placebo) and chronic pancreatitis (0 omarigliptin versus 1 placebo) are infrequent events. The results of this study could be interpreted to be consistent with the meta-analyses of the three completed CV outcome studies [8], in which a small absolute increase in risk was observed with DPP-4 inhibitor therapy. In this study, pancreatic cancer was also infrequent, but an imbalance in the number of pancreatic cancers was observed (5 patients receiving omarigliptin and 1 patient receiving placebo). The relatively early times of onset of the pancreatic malignancies in the omarigliptin group after initiation of therapy (e.g., 3 cases were diagnosed after <1 year of treatment) is not suggestive of a relationship to study drug.

## Conclusion

In the present study conducted in patients with T2DM and established CV disease, treatment with omarigliptin 25 mg q.w., when used as part of usual diabetes care, did not increase the risk of MACE or the risk of hHF compared with placebo. Omarigliptin was generally well tolerated, with an incidence of hypoglycemia that was consistent with the experience with other DPP-4 inhibitors. These results, conducted in a population of patients with T2DM and established CV disease, while not definitive because of early study termination, nonetheless contribute to the existing dataset supporting the safety of the DPP-4 inhibitor drug class. Further studies are required to determine why differences have been observed across studies conducted with different DPP-4 inhibitors.

## Additional file

**Additional file 1.** Adjudication guidelines for cardiac events.

## Abbreviations

ACS: acute coronary syndrome
AE: adverse event
AGI: alpha-glucosidase inhibitor
AHA: anti-hyperglycemic agent
ALT: alanine aminotransferase
APaT: all-patients-as-treated
AST: aspartate aminotransferase
BMI: body mass index
CABG: coronary artery bypass graft
CAC: Clinical Adjudication Committee
CI: confidence interval
CV: cardiovascular
DPP-4: dipeptidyl peptidase-4
ECG: electrocardiogram
eGFR: estimated glomerular filtration rate
EXAMINE: Examination of Cardiovascular Outcomes with Alogliptin versus Standard of Care
FFSG: fasting-fingerstick glucose
FNF: fatal/nonfatal
HbA1c: glycated hemoglobin
hHF: hospitalization for heart failure
HR: hazard ratio
ITT: intent-to-treat
LS: least squares
MACE: major adverse cardiac events
MedDRA: Medical Dictionary for Regulatory Activities
MDRD: Modification of Diet in Renal Disease
MF: metformin
MI: myocardial infarction
NPH: normal pressure hydrocephalus
PDLC: predefined limits of change
PIO: pioglitazone
PTCA: percutaneous transluminal coronary angioplasty
q.w.: once weekly
QTc: corrected QT interval
SAVOR-TIMI 53: Saxagliptin Assessment of Vascular Outcomes Recorded in patients with diabetes mellitus—Thrombolysis in Myocardial Infarction
SD: standard deviation
SE: standard error
SGLT2i: sodium–glucose cotransporter 2 inhibitor
SOC: system organ class
T2DM: type 2 diabetes mellitus
TECOS: Trial Evaluating Cardiovascular Outcomes with Sitagliptin
ULN: upper limit of normal
US: United States
