# Supplementary material for: A randomized, placebo-controlled study of the cardiovascular safety of the once-weekly DPP-4 inhibitor omarigliptin in patients with type 2 diabetes mellitus
Source: Cardiovasc Diabetol. 2017 Sep 11;16:112. doi: 10.1186/s12933-017-0593-8 (PMC5594521; doi:10.1186/s12933-017-0593-8)
Supplement: Supplementary file 1 — Additional file 1. Adjudication guidelines for cardiac events. [file 12933_2017_593_MOESM1_ESM.docx]

**Additional File 1**

Guidelines for adjudication were excerpted from the following documents:

Clinical Data Interchange Standards Consortium (CDISC) Standardized Definitions for Cardiovascular and Stroke End Point Events in Clinical Trials (2014 Draft).

Thygesen K, Alpert JS, Jaffe AS, Simoons ML, Chaitman BR, White HD. Third Universal Definition of Myocardial Infarction. Circulation. 2012;126:2020-35.

**Adjudication Guidelines for Cardiac Events**

**Criteria for Acute Myocardial Infarction**

- Detection of rise and/or fall of cardiac biomarkers [preferably cardiac troponin (cTn)] with at least one value above the 99^th^ percentile of the upper reference limit (URL) together with evidence of myocardial ischaemia with at least one of the following:
  - Symptoms of ischaemia;
  - New or presumed new significant ST-segment (ST-T) changes or new left bundle branch block (LBBB);
  - Development of pathological Q waves in the ECG;
  - Imaging evidence of new loss of viable myocardium or new regional wall motion abnormality.
  - Identification of an intracoronary thrombus by angiography or autopsy.
- Cardiac death with symptoms suggestive of myocardial ischaemia and presumed new ischaemic ECG changes or new LBBB, but death occurred before cardiac biomarkers were obtained, or before cardiac biomarker values would be increased.
- Percutaneous coronary intervention (PCI) related MI is arbitrarily defined by elevation of cTn values (>5 x 99^th^ percentile URL) in patients with normal baseline values (≤ 99^th^ percentile URL) or a rise of cTn values >20% if the baseline values are elevated and are stable or falling. In addition, either (i) symptoms suggestive of myocardial ischaemia or (ii) new ischaemic ECG changes or (iii) angiographic findings consistent with a procedural complication or (iv) imaging demonstration of new loss of viable myocardium or new regional wall motion abnormality are required.
- Stent thrombosis associated with MI when detected by coronary angiography or autopsy in the setting of myocardial ischaemia and with a rise and/or fall of cardiac biomarker values with at least one value above the 99^th^ percentile URL.
- Coronary artery bypass grafting (CABG) related MI is arbitrarily defined by elevation of cardiac biomarker values (>10 x 99^th^ percentile URL) in patients with normal baseline cTn values (≤ 99^th^ percentile URL). In addition, either (i) new pathological Q waves or new LBBB, or (ii) angiographic documented new graft or new native coronary artery occlusion, or (iii) imaging evidence of new loss of viable myocardium or new regional wall motion abnormality.

**Criteria for Silent Myocardial Infarction (Prior Myocardial Infarction)**

Asymptomatic patients who develop new pathologic Q wave criteria for MI detected during routine ECG follow-up, or reveal evidence of MI by cardiac imaging, that cannot be directly attributed to a coronary revascularization procedure, should be termed ‘silent MI.

For this determination, the ECG must be compared with prior ECGs.

ECG changes associated with silent myocardial infarction (prior myocardial infarction):

- Pathological Q-waves, as defined above
- R-wave ≥ 0.04 seconds in V1-V2 and R/S ≥ 1 with a concordant positive T-wave in the absence of a conduction defect

Any one of the following criteria meets the diagnosis of silent myocardial infarction (prior myocardial infarction):

- Pathological Q waves with or without symptoms in the absence of non-ischaemic causes.
- Imaging evidence of a region of loss of viable myocardium that is thinned and fails to contract, in the absence of a non-ischaemic cause.
- Pathological findings of a prior myocardial infarction.

**Stressor Relationship**

For acute MI, the adjudicator is asked to make a determination as to any possible association between the MI and antecedent “major stressors”, specifically coronary revascularization procedures (i.e. PTCA, atherectomy, stent, or CABG). The following guidelines should be used in making these determinations:

1. Complication of a coronary revascularization procedure: an MI that, in the clinical judgment of the adjudicator, occurred coincident with (or within 72 hours following) and was probably a complication of an antecedent coronary revascularization procedure.

a. By convention, increases of biomarkers greater than 3x99th percentile URL have been designated as defining PCI-related myocardial infarction. A subtype related to a documented stent thrombosis is recognized. Note: For percutaneous coronary interventions (PCI) in patients with normal baseline troponin values, elevations of cardiac biomarkers above the 99th percentile URL are indicative of peri-procedural myocardial necrosis.

b. By convention, increases of biomarkers greater than 5x99th percentile URL plus either new pathological Q waves or new LBBB, or angiographically documented new graft or native coronary artery occlusion, or imaging evidence of new loss of viable myocardium have been designated as defining CABG-related myocardial infarction. Note: For coronary artery bypass grafting (CABG) in patients with normal baseline troponin values, elevations of cardiac biomarkers above the 99th percentile URL are indicative of peri-procedural myocardial necrosis.

**Clinical Classification of Different Types of Myocardial Infarction**

**Type 1: Spontaneous myocardial infarction**

Spontaneous myocardial infarction related to atherosclerotic plaque rupture, ulceration, fissuring, erosion, or dissection with resulting intraluminal thrombus in one or more of the coronary arteries leading to decreased myocardial blood flow or distal platelet emboli with ensuing myocyte necrosis. The patient may have underlying severe CAD but on occasion non-obstructive or no CAD.

**Type 2: Myocardial infarction secondary to an ischaemic imbalance**

In instances of myocardial injury with necrosis where a condition other than CAD contributes to an imbalance between myocardial oxygen supply and/or demand, e.g., coronary endothelial dysfunction, coronary artery spasm, coronary embolism, tachy-/brady-arrhythmias, anaemia, respiratory failure, hypotension, and hypertension with or without LVH.

**Type 3: Myocardial infarction resulting in death when biomarker values are unavailable**

Cardiac death, with symptoms suggestive of myocardial ischaemia, and presumed new ischaemic ECG changes or new LBBB, but death occurring before blood samples could be obtained, before cardiac biomarker could rise, or in rare cases cardiac biomarkers were not collected.

**Type 4a: Myocardial infarction related to percutaneous coronary intervention (PCI)**

Myocardial infarction associated with PCI is arbitrarily defined by elevation of cTn values >5 x 99^th^ percentile URL in patients with normal baseline values (≤ 99^th^ percentile URL) or a rise of cTn values >20% if the baseline values are elevated and are stable or falling. In addition, either (i) symptoms suggestive of myocardial ischaemia or (ii) new ischaemic ECG changes or new LBBB or (iii) angiographic loss of patency of a major coronary artery or a side branch or persistent slow-flow or no‑flow or embolization, or (iv) imaging demonstration of new loss of viable myocardium or new regional wall motion abnormality are required.

**Type 4b: Myocardial infarction related to stent thrombosis:**

Myocardial infarction associated with stent thrombosis is detected by coronary angiography or autopsy in the setting of myocardial ischaemia and with a rise and/or fall of cardiac biomarkers values with at least one value above the 99^th^ percentile URL.

**Type 4c: Myocardial infarction related to percutaneous coronary intervention (PCI) restenosis**

PCI-related MI defined as ≥ 50% stenosis at coronary angiography or a complex lesion associated with a rise and/or fall of cTn values >99th percentile URL and no other significant obstructive CAD of greater severity following: (i) initially successful stent deployment or (ii) dilatation of a coronary artery stenosis with balloon angioplasty (<50%).

**Type 5: Myocardial infarction related to coronary artery bypass grafting (CABG):**

Myocardial infarction associated with CABG is arbitrarily defined by elevation of cardiac biomarker values >10 x 99^th^ percentile URL) in patients with normal baseline cTn values (≤ 99^th^ percentile URL). In addition, either (i) new pathological Q waves or new LBBB, or (ii) angiographic documented new graft or new native coronary artery occlusion, or (iii) imaging evidence of new loss of viable myocardium or new regional wall motion abnormality.

**Unstable Angina Requiring Hospitalization**

1. Ischemic discomfort (angina, or symptoms thought to be equivalent ≥ minutes in duration occurring at rest or in an accelerating pattern of angina with frequent episodes associated with progressively decreased exercise capacity.

**AND**

2. Prompting an unscheduled hospitalization **within 24 hours** of the most recent symptoms. Hospitalization is defined as an admission to an inpatient unit or a visit to an emergency department that results in at least a 24 hour stay (or a change in calendar date if the time of admission/discharge is not available).

**AND**

3. At least one of the following:

a. New or worsening ST or T wave changes on resting ECG (in the absence of confounders, such as LBBB or LVH)

• ST elevation

New ST elevation at the J point in two contiguous leads with the cut-off points≥ 0.1 316 mV in all leads other than leads V2-V3 where the following cut-points apply: 317 ≥ 0.2 mV in men ≥ 40 years (≥ 0.25 mV in men < 40 years) or 318 ≥ 0.15 mV in women..

• ST depression and T-wave changes

New horizontal or down-sloping ST depression ≥ 0.05 mV in two contiguous lead; and/or new T inversion ≥ 0.3 mV in two contiguous leads with prominent R wave or T/S ratio >1.

b. Definite evidence of inducible myocardial ischemia as demonstrated by:

• an early positive exercise stress test, defined as ST elevation or ≥ 2 mm ST depression prior to 5 mets **OR**

• stress echocardiography (reversible wall motion abnormality) **OR**

• myocardial scintigraphy (reversible perfusion defect), **OR**

• MRI (myocardial perfusion deficit under pharmacologic stress).

And believed to be responsible for the myocardial ischemic symptoms/signs.

c. Angiographic evidence of new or worsened ≥ 70% lesion (≥ 50% for left main lesion) and/or thrombus in an epicardial coronary artery that is believed to be responsible for the myocardial ischemic symptoms/signs.

d. Need for coronary revascularization procedure (PCI or CABG) for the presumed culprit lesion(s). This criterion would be fulfilled if revascularization was undertaken during the unscheduled hospitalization, or subsequent to transfer to another institution without interceding home discharge.

**AND**

4. Negative cardiac biomarkers and no evidence of acute MI

**General Considerations**

1. Escalation of pharmacotherapy for ischemia, such as intravenous nitrates or increasing dosages of β-blockers, should be considered supportive of the diagnosis of unstable angina. However, a typical presentation and admission to the hospital with escalation of pharmacotherapy, without any of the additional findings listed under category 3, would be insufficient to support classification as hospitalization for unstable angina.

2. If subjects are admitted with suspected unstable angina, and subsequent testing reveals a non-cardiac or non-ischemic etiology, this event should not be recorded as hospitalization for unstable angina. Potential ischemic events meeting the criteria for myocardial infarction should not be adjudicated as unstable angina.

3. Planned hospitalization or rehospitalization for performance of an elective revascularization in patients who do not fulfill the criteria for unstable angina should not be considered a hospitalization for unstable angina. For example,

• Hospitalization of a patient with stable exertional angina for coronary angiography and PCI that is prompted by a positive outpatient stress test should not be considered hospitalization for unstable angina.

• Rehospitalization of a patient meeting the criteria for unstable angina who was stabilized, discharged, and subsequently readmitted for revascularization, does not constitute a second hospitalization for unstable angina.

4. A patient who undergoes an elective catheterization where incidental coronary artery disease is found and who subsequently undergoes coronary revascularization will not be considered as meeting the hospitalization for unstable angina end point.

**Cardiovascular death** includes death resulting from an acute myocardial infarction (MI), sudden cardiac death, death due to heart failure (HF), death due to stroke, death due to cardiovascular (CV) procedures, death due to CV hemorrhage, and death due to other CV causes.

Classifying CV mortality more specifically (MI, sudden death etc.) is usually not needed for outcome trials. However, such classification is difficult because the classifications refer both to underlying cause (e.g., acute MI) and to mode of death (sudden/arrhythmic, progression of HF), and they overlap substantially. The following definitions can, however, be used if desired.

**Death due to Acute Myocardial Infarction** refers to a death by any cardiovascular mechanism (e.g., arrhythmia, sudden death, heart failure, stroke, pulmonary embolus, peripheral arterial disease) ≤ 30 days after a MI related to the immediate consequences of the MI, such as progressive heart failure or recalcitrant arrhythmia. We note that there may be assessable mechanisms of cardiovascular death during this time period, but for simplicity, if the cardiovascular death occurs ≤ 30 days of the myocardial infarction, it will be considered a death due to myocardial infarction.

Acute MI should be verified to the extent possible by the diagnostic criteria outlined for acute MI (see Chapter 4) or by autopsy findings showing recent MI or recent coronary thrombosis.

Death resulting from a procedure to treat a MI (percutaneous coronary intervention (PCI), coronary artery bypass graft surgery (CABG), or to treat a complication resulting from MI, should also be considered death due to acute MI.

Death resulting from an elective coronary procedure to treat myocardial ischemia (i.e. chronic stable angina) or death due to a MI that occurs as a direct consequence of a CV investigation/ procedure/ operation should be considered as a death due to a CV procedure.

**Sudden Cardiac Death** refers to a death that occurs unexpectedly, not following an acute MI, and includes the following deaths:

1. Death witnessed and occurring without new or worsening symptoms

2. Death witnessed within 60 minutes of the onset of new or worsening cardiac symptoms, unless the symptoms suggest acute MI

3. Death witnessed and attributed to an identified arrhythmia (e.g., captured on an electrocardiographic (ECG) recording, witnessed on a monitor, or unwitnessed but found on implantable cardioverter-defibrillator review)

4. Death after unsuccessful resuscitation from cardiac arrest (e.g., implantable cardioverter defibrillator (ICD) unresponsive sudden cardiac death, pulseless electrical activity arrest)

5. Death after successful resuscitation from cardiac arrest and without identification of a specific cardiac or non-cardiac etiology

6. Unwitnessed death in a subject seen alive and clinically stable ≤ 24 hours prior to being found dead without any evidence supporting a specific non-cardiovascular cause of death (information regarding the patient’s clinical status preceding death should be provided, if available)

**Death due to Heart Failure** refers to a death in association with clinically worsening symptoms and/or signs of heart failure regardless of HF etiology (see Chapter 7). Deaths due to heart failure can have various etiologies, including single or recurrent myocardial infarctions, ischemic or non-ischemic cardiomyopathy, hypertension, or valvular disease.

**Death due to Stroke** refers to death after a stroke that is either a direct consequence of the stroke or a complication of the stroke. Acute stroke should be verified to the extent possible by the diagnostic criteria outlined for stroke (**These events will be adjudicated by the Cerebrovascular Subcommittee**).

**Death due to Cardiovascular Procedures** refers to death caused by the immediate complications of a cardiac procedure.

**Non-cardiovascular death** is defined as any death with a specific cause that is not thought to be cardiovascular in nature. The following is a list of potential non-CV causes of death. The reviewer will not be asked to make a judgment regarding the specific cause of a non-CV death.

• Pulmonary

• Renal

• Gastrointestinal

• Hepatobiliary

• Pancreatic

• Infection (includes sepsis)

• Inflammatory (e.g., Systemic Inflammatory Response Syndrome (SIRS) / Immune (including autoimmune) (may include anaphylaxis from environmental (e.g., food) allergies)

• Hemorrhage that is neither cardiovascular bleeding or a stroke

• Non-CV procedure or surgery

• Trauma

• Suicide

• Non-prescription drug reaction or overdose

• Prescription drug reaction or overdose (may include anaphylaxis)

• Neurological (non-cardiovascular)

• Malignancy

**Undetermined Cause of Death** refers to a death not attributable to one of the above categories of CV death or to a non-CV cause. Inability to classify the cause of death may be due to lack of information (e.g., the only available information is “patient died”) or when there is insufficient supporting information or detail to assign the cause of death. In general, most deaths should be classifiable as CV or non-CV, and the use of this category of death, therefore, should be discouraged and should apply to few patients in well-run clinical trials.

A common analytic approach for cause of death analyses is to assume that all undetermined cases are included in the CV category (e.g., presumed CV death, specifically “death due to other CV causes”).

For this program, based on the fact that most of the subjects have a history of CV disease, and all have T2DM, undetermined cause of death will be presumed to be a CV death and counted as a CV death. This option will be included as a category of a fatal event.

**Adjudication Guidelines for Heart Failure**

A **Heart Failure Event** includes hospitalization for heart failure and may include urgent outpatient visits. HF hospitalizations should remain delineated from urgent visits. If urgent visits are included in the HF event endpoint, the number of urgent visits needs to be explicitly presented separately from the hospitalizations.

**A Heart Failure Hospitalization** is defined as an event that meets **ALL** of the following criteria:

1) The patient is admitted to the hospital with a primary diagnosis of HF

2) The patient’s length-of-stay in hospital extends for at least 24 hours (or a change in calendar date if the hospital admission and discharge times are unavailable)

3) The patient exhibits documented new or worsening symptoms due to HF on presentation, including **at least ONE** of the following:

a. Dyspnea (dyspnea with exertion, dyspnea at rest, orthopnea, paroxysmal nocturnal dyspnea)

b. Decreased exercise tolerance

c. Fatigue

d. Other symptoms of worsened end-organ perfusion or volume overload (must be

specified and described by the protocol)

4) The patient has objective evidence of new or worsening HF, consisting of **at least TWO** physical examination findings **OR** one physical examination finding and **at least ONE** laboratory criterion), including:

a. Physical examination findings considered to be due to heart failure, including new or worsened:

i. Peripheral edema

ii. Increasing abdominal distention or ascites (in the absence of primary hepatic disease)

iii. Pulmonary rales/crackles/crepitations

iv. Increased jugular venous pressure and/or hepatojugular reflux

v. S3 gallop

vi. Clinically significant or rapid weight gain thought to be related to fluid retention

b. Laboratory evidence of new or worsening HF, if obtained within 24 hours of presentation, including:

i. Increased B-type natriuretic peptide (BNP)/ N-terminal pro-BNP (NT-proBNP) concentrations consistent with decompensation of heart failure (such as BNP > 500 pg/mL or NT-proBNP > 2,000 pg/mL). In patients with chronically elevated natriuretic peptides, a significant increase should be noted above baseline.

ii. Radiological evidence of pulmonary congestion

iii. Non-invasive diagnostic evidence of clinically significant elevated left- or right-sided ventricular filling pressure or low cardiac output. For example, echocardiographic criteria could include: E/e’ > 15 or D-dominant pulmonary venous inflow pattern, plethoric inferior vena cava with minimal collapse on inspiration, or decreased left ventricular outflow tract (LVOT) minute stroke distance (time velocity integral (TVI))

**OR**

iv. Invasive diagnostic evidence with right heart catheterization showing a pulmonary capillary wedge pressure (pulmonary artery occlusion pressure) ≥ 18 mmHg, central venous pressure ≥ 12 mmHg, or a cardiac index < 2.2 L/min/m2

Note: All results from diagnostic tests should be reported, if available, even if they do not meet the above criteria, because they provide important information for the adjudication of these events.

5) The patient receives initiation or intensification of treatment specifically for HF, including **at least ONE** of the following:

a. Augmentation in oral diuretic therapy

b. Intravenous diuretic or vasoactive agent (e.g., inotrope, vasopressor, or vasodilator)

c. Mechanical or surgical intervention, including:

i. Mechanical circulatory support (e.g., intra-aortic balloon pump, ventricular assist device, extracorporeal membrane oxygenation, total artificial heart)

ii. Mechanical fluid removal (e.g., ultrafiltration, hemofiltration, dialysis)

**Adjudication Guidelines for Cerebrovascular Events**

The distinction between a Transient Ischemic Attack and an Ischemic Stroke is the presence of infarction. Persistence of symptoms is an acceptable indicator of acute infarction. The focal neurologic deficit of a Transient Ischemic Attack resolves completely within 24 hours without residua and is not associated with a new abnormality on brain imaging.

Subdural hematomas are intracranial hemorrhagic events and not strokes.

**Transient Ischemic Attack**

Transient ischemic attack (TIA) is defined as a transient episode of focal neurological dysfunction caused by brain, spinal cord, or retinal ischemia, *without* acute infarction.

**Stroke**

**Stroke** is defined as an acute episode of focal or global neurological dysfunction caused by brain, spinal cord, or retinal vascular injury as a result of hemorrhage or infarction.

**Classification:**

**A. Ischemic Stroke**

Ischemic stroke is defined as an acute episode of focal cerebral, spinal, or retinal dysfunction caused by infarction of central nervous system tissue. Hemorrhage may be a consequence of ischemic stroke. In this situation, the stroke is an ischemic stroke with hemorrhagic transformation and not a hemorrhagic stroke.

**B. Hemorrhagic Stroke**

Hemorrhagic stroke is defined as an acute episode of focal or global cerebral or spinal dysfunction caused by intraparenchymal, intraventricular, or subarachnoid hemorrhage.

**C. Undetermined Stroke**

Undetermined stroke is defined as an acute episode of focal or global neurological dysfunction caused by presumed brain, spinal cord, or retinal vascular injury as a result of hemorrhage or infarction but with insufficient information to allow categorization as A or B.

**Additional guideline:**

Ralph L. Sacco, Scott E. Kasner, Joseph P. Broderick et al**.** An Updated Definition of Stroke for the 21st Century: A Statement for Healthcare Professionals From the American Heart Association/American Stroke Association. Stroke. 2013;44:2064-2089

This publication considers the term hemorrhagic stroke as potentially confusing. As excerpted from this publication, *cerebral hemorrhage* is the preferred terminology that encompasses the same diagnoses as those listed above under hemorrhagic stroke.

**Cerebral Hemorrhage**

Hemorrhagic subtypes of stroke, although less common than ischemic stroke and TIA, still have a significant public health impact because of the higher mortality and morbidity associated with them. ICH alone has a nearly 40% case-fatality rate at 30 days. Although it may seem straightforward to define hemorrhagic subtypes of stroke, a number of issues should be considered, including traumatic injury or secondary causes of bleeding, and the impact of newer technologies on the diagnosis of hemorrhage, among others. Hemorrhages in the CNS should be classified as stroke if they are nontraumatic, caused by a vascular event, and result in injury to the CNS. In contrast, traumatic hemorrhages should not be characterized as stroke. The diagnoses included in this section are ICH, SAH (both aneurysmal and nonaneurysmal), and intraventricular hemorrhage.

**Adjudication Guidelines for Peripheral Vascular Events**

For purposes of this classification, peripheral is defined as arterial vascular beds other than cardiac or cerebrovascular, as defined herein. These include the aorta as well as central vascular beds supplying the lungs, intraperitoneal and retroperitoneal organs (e.g., renal, hepatic, mesenteric), the pelvic organs, and the extremities.

**NONFATAL EVENTS**

Peripheral arterial thrombosis or peripheral arterial thromboembolism

Hospitalization for documented signs and symptoms of new onset or accelerating arterial occlusion in a peripheral artery, supported by objective laboratory or surgical evidence.

For events in arteries supplying the extremities, there should be a new onset or accelerating pain with physical findings of ischemia (involving at least one of the following: absent pulse, cool temperature, neurologic deficit, cyanosis or pallor). For events in arteries supplying central structures and extremities, objective laboratory abnormalities include ankle/brachial pressure index or toe/brachial pressure index or duplex Doppler ultrasound or arteriography, CT scan, or magnetic resonance imaging or pathological specimens, or autopsy findings.

**FATAL EVENTS**

**Peripheral arterial thrombosis or peripheral arterial thromboembolism**

A fatal peripheral arterial thrombosis or peripheral atrial thromboembolism is an event as defined above and that is documented to directly result in death.
